# Toxicity and Impact of Silica Nanoparticles on the Configuration of Gut Microbiota in Immunodeficient Mice

**DOI:** 10.3390/microorganisms11051183

**Published:** 2023-04-30

**Authors:** Sana Shabbir, Yanzhou Hu, Xiaoyun He, Kunlun Huang, Wentao Xu

**Affiliations:** 1 College of Food Science and Nutritional Engineering, China Agricultural University, Beijing 100083, China; 2 Key Laboratory of Precision Nutrition and Food Quality, Department of Nutrition and Health, China Agricultural University, Beijing 100083, China; 3 Key Laboratory of Safety Assessment of Genetically Modified Organism (Food Safety) (MOA), College of Food Science and Nutritional Engineering, China Agricultural University, Beijing 100083, China

**Keywords:** Cy-immunodeficiency, silica nanoparticles, intragastric gavage, immune dysfunctioning, RNA sequencing, microbial diversity

## Abstract

Nanoparticles (NPs), having exceptional physicochemical and electrical characteristics with lower toxicity, have evolved as dynamic drug delivery carriers in living organisms. Potentially, the intragastric gavage of silica nanoparticles (SiNPs) affects gut microbiota profiles in immunodeficient mice. In this study, the impact of SiNPs of variable size and dosage was investigated in cyclophosphamide (Cy)-induced immunodeficient mice, specifically on their immune functions and gut microbiota, through physicochemical and metagenomic analysis. SiNPs of different sizes and doses were gavaged to Cy-induced immunodeficient mice for 12 days at an interval of 24 h to investigate their effects on immunological functions and the gut microbiome of mice. Our results showed that SiNPs had no significant toxicological effects on the cellular and hematological activities of immunodeficient mice. Furthermore, after the administration of different levels of SiNPs, no immune dysfunction was found in the immunosuppressed mice groups. However, gut-microbial studies and comparisons of characteristic bacterial diversity and compositions demonstrated that SiNPs significantly affect the abundance of different bacterial communities. LEfSe analysis revealed that SiNPs significantly increased the abundance of *Lactobacillus*, *Sphingomonas*, *Sutterella*, *Akkermansia*, and *Prevotella*, and potentially reduced *Ruminococcus* and *Allobaculum*. Thus, SiNPs significantly regulate and modify the configuration of the gut microbiota in immunodeficient mice. These dynamic variations in the intestinal bacterial community, abundance, and diversity provide new insight into the regulation and administration of silica-based NPs. This would be helpful for the further demonstration of the mechanism of action and prediction of the potential effects of SiNPs.

## 1. Introduction

Nanotechnology has transformed the world by manipulating nanoparticles to address a wide range of contemporary issues, including human health, disease management, and food production [1,2,3,4,5]. Food technologists have faced numerous impediments in increasing the food to meet the need for the massive population of the planet. The nanotechnology synthetically manipulates nanoparticles (NPs) in several ways to address large numbers of overwhelming core issues, such as food processing, food preservation, and animal and plant health. NPs are engineered in multiple ways such as polymeric micelles or liposomes, which are useful to a wide variety of fields including drug/gene delivery [4,5,6,7]. Biomedical applications of NPs, such as targeted drug delivery, hyperthermia photoablation therapy, and bio-imaging systems, have revolutionized biomedical technology [8,9,10,11]. Recently, dynamically novel and potential nanomaterials (NMs), including silica nanoparticles (SiNPs) and mesoporous silica nanoparticles (MSNs), with unique physicochemical and electrical characteristics, have evolved as drug delivery carriers in living organisms [7,11,12,13]. However, careful evaluation for biocompatibility, biodistribution, and agreement for bio-applications require assurance of the safe applications of these nanoparticles in the field of medicine [14,15,16]. Interestingly, SiNPs are generally advantageous in a wide range of fields, including engineering, chemistry, medicine, agriculture, and cosmetics. Instead, there is a lack of knowledge on the adverse effects of the administration of SiNPs. The toxicity of SiNPs depends on their physical and chemical properties such as size, dose, and cell type for the immune system calculations of genotoxicity, influential cytotoxicity, and cell dysfunctions [14,17,18,19]. Extensive development has been achieved in nanotoxicity studies. Still, the biosafety of SiNPs for biomedical applications, and the investigation of their biodistribution and in vivo toxicology is still a matter of consideration [20,21,22,23]. Thus, much elucidation, including on SiNPs’ effects on living organisms, is required. Indeed, it may be life-threatening if the toxicity of SiO_2_ NPs with diameters less than 100 nanometers is not explored, as it can undergo direct immersion to sensitive organs of the body after intravenous administration [24,25]. The impact of NPs on cytotoxicity has major concerns, e.g., the inhibition and modification of cell function followed by serious damages. The effects of geometry, absorbency, and surface charge of SiNPs on cellular toxicity and hemolytic action were previously studied and explored [26,27,28]. Similarly, sub-acute toxicity by the venous administration of silver NPs (AgNPs) and gold NPs (AuNPs|) in relation to systemic toxicity demonstrated that the mixture did not convince any obvious systemic toxicity [29]. Nevertheless, some probiotic bacterium, *Clostridium butyricum* MIYAIRI (CBM588), increases the richness of gut microbiomes and induces dysbiosis and gut inflammation through anti-inflammatory lipid metabolites [30]. 

The sensible strategy for using NPs is mandatory for biomedical applications. Cyclophosphamide (Cy) is used for symbolic chemotherapeutic discharge bacterial and infection translocations produced by mucosal barrier chemotherapy combined with treatment with antibiotics. Gastrointestinal side effects and hepatotoxicity are often associated with Cy due to its characteristics as an immunosuppressor [31,32,33,34]. Modern mechanisms have revealed the remedial possessions of Cy to be determined by abdominal microbes since the action of Cy reduced *Bacteroides* proportions but increased *Firmicutes* [35]. Likewise, Cy clearly enhanced microorganisms in the stool and triggered a “leaky” gut [36,37,38,39]. 

A large number of experimentations have discovered the impact of orally administered nanostructured Silica (SiO_2_) on a regular basis in various doses as a food additive on the immune functions and gut microbiota of rats through its physicochemical and biological properties. Inhaled silica dust plays an important role in tuberculosis (TB) growth and progress [40,41,42,43]. The link between the size of amorphous SiO_2_ and inflammatory activity was addressed irrespective of diameters. No correlation was reported in SiNPs’ accumulation and colonic inflammation correlation [41,44,45]. We designed this study in order to investigate the effect of SiNPs on the gut microbiome in the immunodeficient mice model. In this study, we evaluated the safety assessment of SiNPs on Cy-induced immunodeficient mice. In addition, this study aimed to calculate the toxicity of SiNPs and alleviate the impact of the gut microbiome of mice at the phylum and gene levels by subjecting them to SiNPs of different sizes and dosages at different intervals.

## 2. Materials and Methods

### 2.1. Chemicals and Reagents 

All chemicals and experimental ingredients used in this study for the preparation of mesoporous silica were of analytical grade. These chemicals and reagents, including tetraethyl orthosilicate (TEOS, ≥99%), cetyl trimethyl ammonium chloride (CTAC, ≥98%), triethanol (TEA, ≥99%), and sodium hydroxide (NaOH, ≥99.9%) were purchased from Sigma-Aldrich (Saint Louis, MO, USA).

### 2.2. Manufacturing of NMs

MSNs, such as mobil composition of matter 41 (MCM-41), are the most classic and widely used controlled drug delivery systems in mouse model experimentation. A typical method for its preparation was achieved by the modification of wet synthesis [46,47]. The wet synthesis procedure can be summarized as: In 20 mL of double-distilled water, 2 g of CTAC and 0.08 g of TEA were dissolved and magnetically stirred for 1 h at 95 °C. Briefly, 1.5 mL of TEOS was added dropwise (0.25 mL·min^−1^) and stirred for 1 h to observe the chemical reaction (formation of a milky solution) and allowed for a certain time to complete silica condensation. The collected MSNs were isolated by centrifugation (10,000 rpm, 10 min), washed with ethanol to remove the residuals of the reactions, and dried at 60 °C for 24 h. For the extraction of the surfactant CTAC, the collected MSNs were added to ethanol salt solution (15 mL MSNs into 100 mL ethanol), ultrasonic treatment was given for 2 h, and it was stirred at 70 °C for 24 h. This step was repeated twice to completely remove the surfactant (CTAC). Finally, centrifugal sediments were washed with ethanol, dispersed in ethanol, and stored in a refrigerator. For certain analyses, the prepared particles were dried under vacuum in an oven at 40 °C for 48 h.

### 2.3. Animals and Treatments

Six-week-old mice (Balb/c) were bought from Vital River Laboratories, Beijing, China, and allowed to adapt for one week before the start of the experimental trial. The room temperature was 22 ± 2 °C with a relative humidity of 55 ± 10% with a 12 h light/dark cycle in a specific pathogen-free (SPF) animal room. After 1 week, the mice’s weights ranged between 20 and 22 g. They were divided into 8 groups, each group consisting of 3 to 4 mice. These groups were named as control groups, CK-1 and CK-2, and Cy immunodeficiency model groups, Cy-1, Cy-2, Cy-silica-small-low (SiO_2_ size: 15 nm, dose: 40 mg/kg per day) (Si-SL), Cy-silica-small-high (SiO_2_ size: 15 nm, dose: 80 mg/kg per day) (Si-SH), Cy-silica-big-low (SiO_2_ size: 50 nm, dose: 40 mg/kg per day) (Si-BL), and Cy-silica-big-high (SiO_2_: 50 nm, dose: 80 mg/kg per day) (Si-BH). Prior to the intragastric gavage of SiNPs, intraperitoneal injections of Cy bought from Shanghai Aladdin Biochemical Technology Co., Ltd. at the dose of 80 mg/kg per day were injected for 3 days to induce immunodeficiency in the mice. The weight was taken every day. After immunosuppression was completed, the control group (CK-1) and the model group (Cy-1) were slaughtered to prove the success of the immunodeficiency model. The body weight, immune organ weight (thymus and spleen), and the number of blood-routine immune cells were determined. Then, the intragastric gavage of SiNPs and other drugs was given every 24 h for 12 days. The protocols of the study were approved by the Animal Care Committee of China Agricultural University and followed the guidelines of the Care and Use of Laboratory Animals, Beijing, China.

### 2.4. Sampling for Silica Toxicity Analysis 

To detect any systemic dysfunction in mice after the application of SiNPs, we examined blood biochemical changes. Blood samples were collected in duplicate, and enough blood was collected before the mice were sacrificed from the ocular vein of the mice after the exposure to SiNPs. These samples were refrigerated at −20 °C and submitted for testing. To study the physiochemical and toxicological effect of SiNPs, all mice were sacrificed after blood withdrawal, and major organs, such as the thymus, spleen, liver, kidneys, and testes, were harvested. These organs were fixed in 10% formalin and subjected to subsequent examinations [48,49].

### 2.5. Toxicity Analysis after the Exposure of SiNPs 

In order to determine the toxicity of SiNPs in mice, the physiological parameters of different organs were calculated to examine the cellular effect or pathological events. Blood samples were subjected to biochemical analysis. Complete blood counts (CBC) were analyzed by a hematology analyzer, HEMAVET^®^950FS (Drew scientific, Dusseldorf, Germany). In order to obtain serum, the blood samples were centrifuged at 30,000 rpm for 15 min. The serum biochemical profiles for alanine aminotransferase (ALT), aspartate aminotransferase (AST), alkaline phosphatase (ALP), albumin (ALB), total protein (TP) uric acid (UA), blood urea nitrogen (BUN), and creatinine (CRE) were analyzed using an RA-1000 autoanalyzer (Technicon, USA). Immunoglobulin (Ig) was measured using commercially available kits (IgG/A/M kit) according to the manufacturers’ instructions. In addition, inflammatory cytokines in the serum and ileum of modeled mice were determined through the rat enzyme-linked immunosorbent assay (ELISA). The company whose ELISA kit we used is Sinoukbio (Beijing, China) [50,51].

### 2.6. Feces Sampling for Gut Microbiome Analysis

For this purpose, the mice were isolated and placed in separate cages, and fresh stool samples (approximately 100 mg) were collected from the mice at various time intervals and transferred into 2 mL aseptic centrifuge tubes using sterilized high-pressure forceps. These samples were snap-frozen in liquid nitrogen before storage at −80 °C until DNA extraction. 

### 2.7. DNA Extraction and 16 Metagenomic Sequencing

DNA samples were extracted using a fecal DNA isolation kit (FUDEAN, Beijing, China) according to the instructions of the manufacturer. The quality of DNA (260/280 ratio) was determined using agarose gel electrophoresis and a NanoDrop ND-1000 UV-Vis Spectrophotometer (NanoDrop Technologies Inc., Wilmington, DE.) The V3-V4 region of the 16S rRNA was amplified by PCR using universal primers (338 forward: 5′-ACTCCTACGGGAGGCAGCAG-3′; 806 reverse: 5′-GGACTACHVGGGTWTCTAAT-3′). The DNA samples were sent to Nuo he source bio Mdt InfoTech Ltd. for metagenomic sequencing. 

### 2.8. Gut Microbiome Community Analysis

The raw data of the sequencing were analyzed by the Oracle VM Virtual Box system. The sequencing was performed in a HiSeq platform (Illumina, San Diego, CA, USA) at Novogene Bioinformatics Institute (Beijing, China). The raw reads were spliced and filtered to obtain clean reads. The operational taxonomic unit (OTU) clustering was performed by Uparse software (Uparse v7.0.1001), and sequences with ≥97% similarity were clustered to the same OTUs. Taxonomic annotation was conducted using the ribosomal database project (RDP) classifier (Version 2.2). Then, the operational taxonomic units (OTUs) were investigated for the analysis of the overall change and diversity of the intestinal microbial community. Multivariate/non-metric multidimensional scaling (NMDS), metric multidimensional scaling (MDS), and alpha diversity and beta diversity analysis by PAST software were used to analyze the differences between the samples. A heat map was created with Heml Software (version 1.0.3.7). Moreover, to predict significant changes in the relative abundance of the microbial taxa, linear discriminant analysis (LDA) effect size (LEfSe) was performed [52,53,54,55].

### 2.9. Statistical Analysis

A single-factor analysis of variance (ANOVA) followed by a two-tailed Student’s t-test were used for comparisons. Almost all data were presented as means ± SEM. Significant differences were considered when *p* < 0.05. Graph-Pad Prism 5 (Graph Pad Software, California, America) was used for data analysis. Species diversity in each sample based on different indices and rarefied “OUT” counts were analyzed using R software (Version 2.15.3). In this study, an alpha significance level of 0.05 and an effect size threshold of 3 were used as cut-off values. A value of *p <* 0.05 was deemed significant in all statistical tests. The operational taxonomic unit (OTU) clustering was performed by Uparse software (Uparse v7.0.1001), and sequences with ≥97% similarity were clustered to the same OTUs. Taxonomic annotation was conducted using the ribosomal database project (RDP) classifier (Version 2.2).

## 3. Results

### 3.1. Analysis of SiNPs on Cellular and Hematological Activities of Immunodeficient Mice

**Figure 1:**
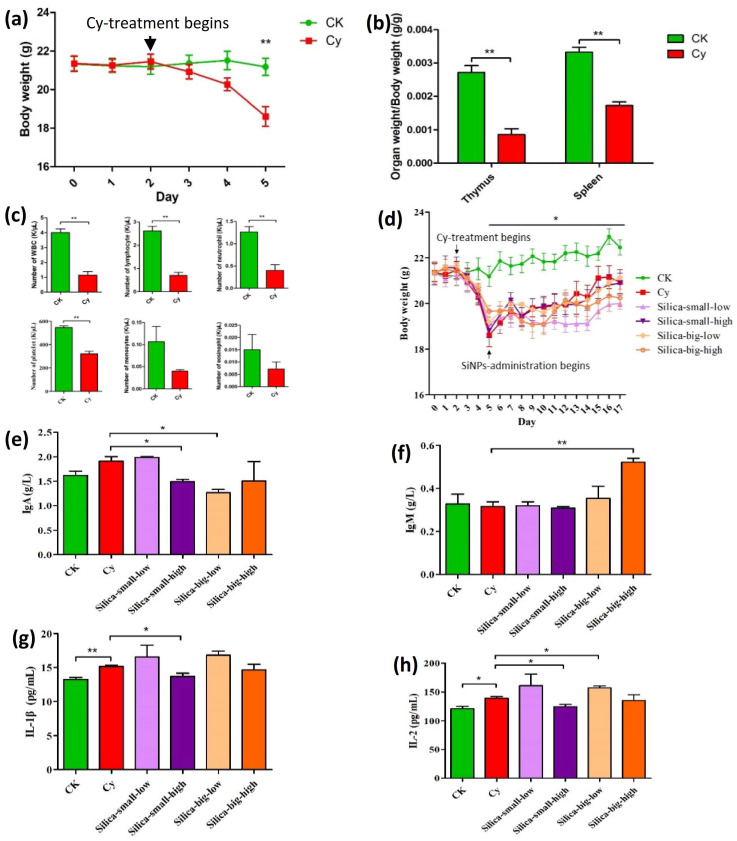
Toxicological analysis of Cy-immunodeficiency model (mice) gavaged different levels of SiNPS. (**a**) Showing body weight after 3 days of consecutive Cy-injection treatment, and (**b**) organ coefficient (organ weight/body weight) of immune organs (thymus and spleen). (**c**) The total number of white blood cells (WBC), lymphocytes, neutrophils, and platelets were significantly reduced, whereas monocytes and eosinophils were reduced but not significantly. CK represents the control group, whereas Cy denotes the Cy-induced immunodeficiency model (mice). CK represents the control group, whereas Cy denotes the Cy-induced immunodeficiency model (mice). (**d**) A comparative analysis of SiNPs on mice, administered with different dosages, such as Cy-silica-small-low (SiO_2_ size: 15 nm, dose: 40 mg/kg per day), Cy-silica-small-high (SiO_2_ size: 15 nm, dose: 80 mg/kg per day), Cy-silica-big-low (SiO_2_ size: 50 nm, dose: 40 mg/kg per day), and Cy-silica-big-high (SiO_2_: 50 nm, dose: 80 mg/kg per day). (**e**) The serum immunoglobulin analysis revealed significant changes in immunoglobulin A (IgA) and (**f**) immunoglobulin M (IgM). Serum inflammatory cytokines analysis showed an increased level of (**g**) IL-1β and (**h**) IL-2 in the model groups than that of the immunodeficiency group. *, statistically significant (*p* ≤ 0.05); **, statistically significant (*p* ≤ 0.01).

In order to analyze the cellular and hematological impact of SiNPs, an immunodeficiency model (mice) was successfully created; significant weight loss is a most natural indicator of the Cy-immunodeficiency model. The body weight and organ coefficient of immune organs (thymus and spleen) in the model group were significantly decreased (Figure 1a,b). Furthermore, the immune cells, the total number of white blood cells (WBC), lymphocytes, neutrophils, and platelets were significantly reduced, whereas monocytes and eosinophils, were reduced but not significantly (Figure 1c).

After the immunosuppression modeling, the mice were administered with different dosages of SiNPs, such as Cy-silica-small-low (SiO_2_ size: 15 nm, dose: 40 mg/kg per day), Cy-silica-small-high (SiO_2_ size: 15 nm, dose: 80 mg/kg per day), Cy-silica-big-low (SiO_2_ size: 50 nm, dose: 40 mg/kg per day), and Cy-silica-big-high (SiO_2_: 50 nm, dose: 80 mg/kg per day). The body weight of the mice decreased significantly, and then the body weight slowly increased. There was a significant difference between the Cy model group and the CK group, but there was no significant difference between the drug-administered group and the model group (Figure 1d). In the case of immune organs (thymus and spleen), there were significant changes between the immunodeficient Cy model group and the CK group. The thymus became smaller, and the spleen became larger in the immunodeficient mice. It may be due to infection and immunodeficiency. The thymus of the Si-SL group was slightly smaller than that of the Si-SH group. No difference was seen for the thymus between the Si-BL and Si-BH groups. The Si-BL group was affective and increased spleen size as compared to Si-BH concentration. That means size does not affect it, but it does affect it when the dose level is decreased. The size of the thymus also decreased and the spleen size increased. However, there were no significant differences in the weight of immune organs between each administration group and the model group. For other organs, such as the kidneys and testes, low-dose 15 nm silica and high-dose 50 nm silica reduced the kidney organ coefficient; the model group and CK compared with the decrease in testicular organ coefficients (Figure S1). Moreover, we also found that immune cells, hemoglobin, and other blood indicators were not significantly affected by different doses of SiNPs. However, the average number of platelets and the hemoglobin contents were higher in the high-dose 50 nm silica-administered group than that in the model group (Figure S2). The serum biochemical analysis revealed that each treatment had no significant effect on the liver and/or renal function because the levels of ALT, AST, ALP, ALB, TP, UA, BUN, and CRE were not affected by SiNPs (Figure S3). Thus, it was proved that renal function was not affected. The serum immunoglobulin A (IgA) levels were significantly decreased in the high-dose 15 nm and low-dose 50 nm silica nanoparticle groups (Figure 1e), whereas the immunoglobulin M (IgM) levels were significantly higher in the group that received high-dose 50 nm silica nanoparticles (Figure 1f). No other significant differences were found in immunoglobulin G (IgG). In addition, serum inflammatory cytokines IL-1β and IL-2 were increased in the model groups compared to that of the immunodeficiency group. That may be due to an increase in the inflammation of cytokines. However, inflammatory cytokines in the ileum did not differ significantly in each treatment of silica particles (Figure S4).

### 3.2. Effect of SiNPs on Gut Microbiome in Immunodeficient Mice 

#### 3.2.1. Intestinal Microbiota Profiling in Immunodeficient Mice Prior to SiNP Treatments

**Figure 2:**
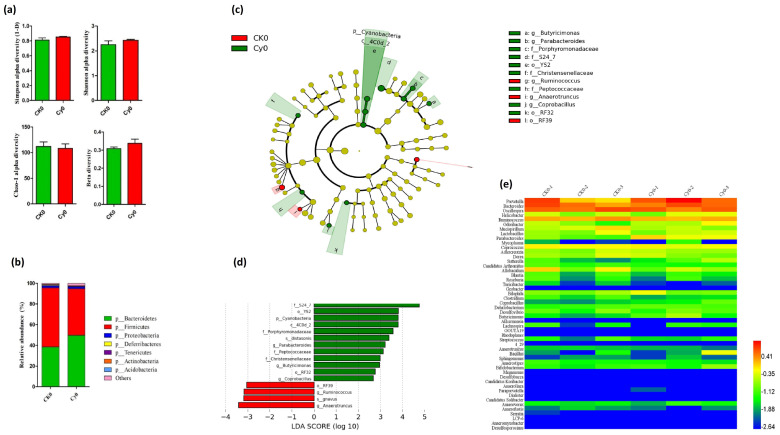
The gut bacterial community analysis between control CK0 group and model Cy0 group were determined through 16S ribosomal RNA (rRNA) gene sequencing. (**a**) Alpha (Chao-1, Simpson, and Shannon) and beta diversity between these two groups showed no significant differences (**b**) but with differences in Bacteroides and Firmicutes at the phylum level. Statistically, different levels of bacterial community composition were found between the aforementioned groups through LDA (green bar indicating the abundance of intestinal bacteria in the Cy0 group and red bar showing the abundance of intestinal bacteria in the (**c**) CK0 group and (**d**) LEfSe). (**e**) Heatmap also shows the differences in the levels of intestinal bacteria between the two groups.

Prior to determining the effect of SiNPs, we performed intestinal microbiome analysis in the control CK0 and Cy-induced immunodeficient model Cy0 groups. The results showed that there were no significant differences in the intestinal microbiome diversity between the control group (CK0) group and the immunodeficiency group (Cy0) (Figure 2a). However, the relative abundance of the Bacteroides phylum increased, and the Firmicutes phylum decreased in the Cy0 group (Figure 2b). LDA coupled with LEfSe analysis also showed statistically and biologically consistent differences from the phylum to the genus level. The analysis showed the abundance of intestinal bacteria in the Cy0 group (green bar) that was significantly higher than that of the CK0 group (red bar). In addition, it also expressed the taxonomic relationship between differentially abundant intestinal bacteria. The LEfSe analysis after immunosuppression indicated that CK0-dominant genera were Ruminococus and Anaerotruncus, and Cy0-dominant genera were Parabacteroides, Butyricimonas, and Coprobacillus (Figure 2c,d).

#### 3.2.2. Gut Microbiota Profiling in Immunodeficient Mice after Gavage of SiNPs

To describe the effect of different doses of SiNPs on gut microbiota in immunodeficient mice, the intestinal bacterial community, abundance, and diversity were analyzed after the intragastric gavage of SiNPs at intervals of 6 and 12 days.

#### 3.2.3. Gut Microbial Diversity and Abundance at Phylum Level

**Figure 3:**
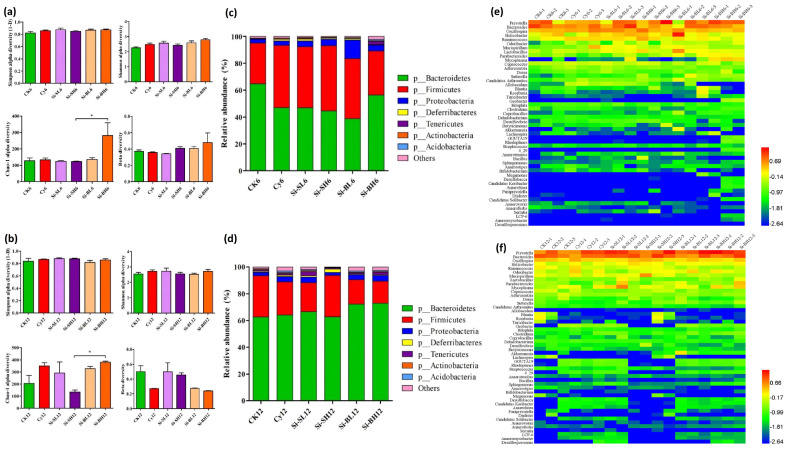
The effect of different levels of doses of SiNPs on gut microbiota in immunodeficient mice. (**a**,**b**) These different levels of doses significantly affect the alpha and beta diversity of the gut microbial community after 6 and 12 days of SiNP gavage. (**c**,**d**) At the same treatments, gut microbiota showed different levels of abundance at the phylum level. (**e**,**f**) Heatmap also shows the differences in the levels of intestinal bacteria after 6 and 12 days of SiNP gavage. *, statistically significant (*p* ≤ 0.05).

The results showed that there were no significant differences between the Simpson and Shannon alpha diversity of the bacterial community, but in the Chao-1 alpha diversity, significant differences were found between the Si-SH6 and Si-BH6 model groups after 6 days of SiNP gavage. In the case of beta diversity, however, a minor difference was found between Si-BH6 and the other SiNP-treated groups of immunodeficient mice (Figure 3a). 

Similarly, after 12 days, Chao-1 alpha diversity was significantly higher in the Si-BL12 and Si-BH12 groups than in Si-SH12. Meanwhile, the Simpson and Shannon alpha diversity did not show significant differences, but the beta diversity showed major differences among the Si-BL12, Si-BH12, Si-SL12, and Si-SH12 model groups (Figure 3b). Moreover, the microbial relative abundance analysis demonstrated that the Bacteroides phylum gradually decreased and the Firmicutes and Proteobacteria phyla increased in the modeled groups (Si-SL6, Si-SH6, and Si-BL6) after 6 days of SiNP treatment. Conversely, in the Si-BH modeled group, Bacteroides and Tenericutes increased, and Firmicutes and Proteobacteria decreased (Figure 3c). After the 12 days of SiNP gavage, the results showed that the level of the Bacteroides phylum increased in all immunodeficient mice modeled groups (Si-SL12, Si-BL12, and Si-BH12), except Si-SH12, where the relative abundance of the Firmicutes phylum was higher (Figure 3d). Thus, it is obvious from the aforementioned results that dose size and treatment days significantly affect the diversity and relative abundance of gut microbiota. Additionally, the Chao-1 alpha diversity was significantly higher after 12 days of treatment. Similar trends were observed in the relative abundance of the Bacteroides phylum after 6 and 12 days at both high and low levels of doses. On the other hand, SiNPs of large size had different trends after 6 days of gavage for the phylum Proteobacteria because, at low doses, a higher abundance of the Proteobacteria was found. After 12 days, SiNPs of varying sizes and doses have a discernible effect on the Deferribacters and Tenericutes phyla (Figure 3). It was concluded that SiNPs of small size at higher and lower doses changed the composition of microorganisms in the gut of mice after 12 days.

#### 3.2.4. Comparison of Abundant Intestinal Bacteria and Taxonomic Relationship

**Figure 4:**
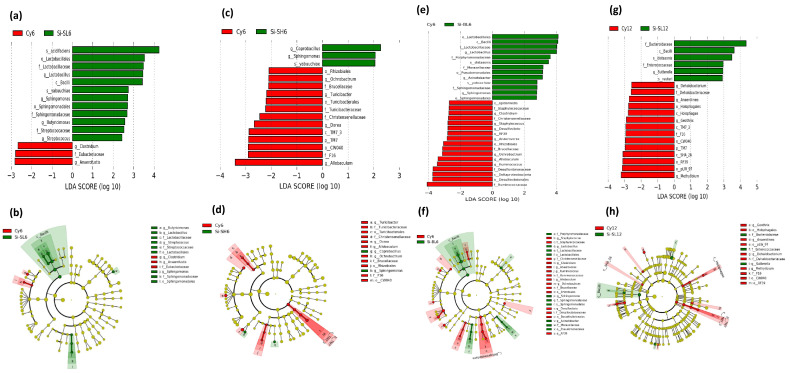
LEfSe analysis demonstrating the comparison of different bacterial genera that were sinificantly varied in abundance at different levels and days of SiNP treatments. As shown, intestinal bacteria significantly differed between the immunodeficient groups and SiNP treatment groups at different levels, such as the (**a**,**b**) Si-SL6-treated group, the (**c**,**d**) Si-SH6-treated group, as well as the (**e**,**f**) Si-BL6-treated group, after 6 days of treatment with SiNPs. Moreover, (**g**,**h**) bacterial abundance was significantly changed after 12 days of SiNP treatments.

The LEfSe analysis was applied to compare the differentially abundant intestinal bacteria at the genus level. The specific difference in intestinal bacteria was found through LEfSe analysis, in which the Cy groups and the SiNP-administered groups were compared and interconnected after Cy injections and after 6 and 12 days of SiNP treatment. This analysis was used to identify dominant bacteria in each group of mice, demonstrating the relationship between intestinal bacteria species with marked differential abundance (Table 1). The findings showed that after 6 days of SiNP administration, the abundance of intestinal bacteria varies with each level of treatment. Abundant genera in the CK6 control group, Prevotella, and Anaerofustis, were found, whereas, in the Cy6-immunodeficient group, the dominant genera were Oscillospira, Anaeroplasma, Allobaculum, Ruminococcus, Odoribacter, Sutterella, and Coprococus. Similar differences in gut microbiota at the genus level were compared by LEfSe analysis (Figure S5). Further analysis between the Cy6-immunodeficient group and different levels of SiNP-treated groups demonstrated significant variation in different abundant genera. For example, those such as Clostridium and Anaerofustis were found in the Cy6-immunodeficient group; however, the dominant genera in the Si-SL6-treated group were Lactobacillus, Sphingomonas, Butyricimonas, and Streptococcus. Similarly, Coprobacillus and Sphingomonas were abundant in the Si-SH6-treated mice, Lactobacillus, Acinetobacter, and Sphingomonas in the Si-BL6-treated mice, and Prevotella, Mycoplasma, Parabacteroides, Lactobacillus, Proteus, Mycobacterium, Sutterella, Paraprevotella, Coprobacillus, and Turicibacter were significantly abundant in the Si-BH6-treated immunodeficient mice group (Table 1 and Figure 4a–f). 

After 12 days of SiNP treatment, the LEfSe analysis showed that the CK12 control group contained Streptococcus, Anaerostipes, Sutterella, Dorea, and Bacteroides as abundant genera. On the other hand, Methylibium, Anaeromyxobacter, Desulfococcus, Sphingomonas, Thiobacillus, and Aquicella were found in the Cy12-immunodeficient group. Furthermore, the comparison between the Cy12-immunodeficient mice group and the SiNP-administered groups revealed that the abundance of gut microbiota varies from Dehalobacterium, Anaerofustis, Geothrix, and Methylibium to Anaerostipes, Dehalobacterium, Anaeromyxobacter, Geothrix, Methylibium, Geobacter, Ruminococcus, and Mucispirillum in Cy12-immunodeficient mice as compared to the Si-SL12- and Si-BH12-treated groups, significantly abundant in Sutterella and Prevotella, Sutterella, and Akkermansia, respectively (Figure 4g,h). Interestingly, other groups of mice treated with Si-SH12 and Si-BL12 were also dominant in similar genera (Table 1, Figure S6). Consequently, it is obvious from the results that SiNPs significantly affect the bacterial community. SiNPs significantly increased the abundance of Lactobacillus, Sphingomonas, Sutterella, Akkermansia, and Prevotella and reduced Ruminococcus and Allobaculum. In addition, the effects of Cy on mice immunity and bacterial abundance were validated, which was initially proved by Xu and Zhang in 2015 [35].

## 4. Discussion

The toxicity of SiNPs upon oral exposure was evaluated in immunodeficient mice using physiological and histological changes and blood analysis. In Cy-induced immunosuppressed mice, the body weight, including the organ coefficient of immune organs (thymus and spleen) and immune cells, significantly decreased (Figure 1a–c). However, no significant difference was observed between the drug-administered groups treated with SiNPs of different particle sizes and concentrations (Figure S1), except for significant changes in IgA and IgM. Furthermore, serum inflammatory cytokine analysis showed an increased level of IL-1β and IL-2 in the SiNP-treated model groups compared to that of the immunodeficient and control groups (Figure 1e–h). Consequently, in our study regarding body weight, a major difference was observed between the Cy model group and the CK control group, but no significant differences were observed between the drug-administered groups. This may be due to infection and immunodeficiency [56]. In fact, Cy is the most commonly used drug in autoimmune diseases; it suppresses the growth of mice because Cy is an immunosuppressant, and the related gastrointestinal (GI) lateral effects and hepatotoxicity increase the number of suppressor cells [31,35,57]. In our results, non-significant or mild variations in the physical and behavioral changes in the SiNP-treated mice groups with no mortality were observed. It was clearly comparable to some previous studies which reported that reductions in growth and weight were significantly related to the toxic effects of chemicals [58,59,60]. 

The thymus maintains and initiates an adaptive immune response and it is a specialized primary lymphoid organ of the immune system [61]. Immune organs (thymus and spleen) showed significant changes between the immunodeficient model Cy group and the control CK group. The immunodeficient mice have smaller thymuses and larger spleens. However, there was no significant difference in the weight of immune organs between every SiNP-administered group experiencing different levels of silica doses and concentrations (Figure S1c,d). Similarly, the thymus and spleen indexes vary significantly due to the treatment of Cy, but they had immune stimulatory activity. That means that the immunomodulatory effects of NMs could be beneficial and provide positive effects on the immune response [62]. Commonly, silica induced hepato- and nephrotoxicity that altered oxidative stress and inflammation [63]. In addition, serum inflammatory cytokine analysis revealed that the levels of IL-1β and IL-2 were increased (Figure 1g,h). As Liu et al. (2007) reported, SiNPs induce macrophage cytokines that are involved in silica-associated inflammatory responses [64]. Inflammatory responses, generally less frequent but more lethal, were also detected in the livers of exposed animals [65]. Similarly, renal interstitial fibrosis was induced by high doses of SiNPs [66]. A recent study presented that the intragastric administration of MSNs for 2 weeks improved serum ALP, ALT, and AST and caused inflammation in the spleen and the intestine [67]. Consequently, silica groups have some effect on mice immunoglobulin and inflammatory cytokines. SiNPs have a slight effect on the immune function of immunodeficient mice; similarly, in previous research, different-sized SiNPs caused different immunotoxicity. Small-sized SiNPs showed the most potent in vivo immunotoxicity by suppressing the proliferation of lymphocytes and reducing proinflammatory cytokine production, therefore causing immunosuppression [68]. 

Gut microbiome analysis was conducted after the gastrointestinal exposure of immunodeficient mice with SiNPs of different sizes and concentrations. Diet, antibiotics, and age can change gut microbiota, and many studies have shown the relationship of the microbiota and several diseases and reported some ways to modulate that balance. Microbial relative abundance analysis revealed that the *Firmicutes* and *Proteobacteria* phyla increased and the *Bacteroides* phylum progressively decreased in the modeled groups (Si-SL6, Si-SH6, and Si-BL6) after 6 days of SiNP gavage. Although *Bacteroides* and *Tenericutes* increased, *Firmicutes* and *Proteobacteria* decreased in the Si-BH-modeled group (Figure 3c). After 12 days of SiNP gavage, the microbial abundance of the *Bacteroides* phylum increased in the modeled groups (Si-SL12, Si-BL12, and Si-BH12), except Si-SH12, where the relative abundance of the *Firmicutes* phylum was higher (Figure 3d). Consequently, SiNPs significantly increased the abundance of *Lactobacillus*, *Sphingomonas*, *Sutterella*, *Akkermansia*, and *Prevotella* and reduced *Ruminococcus* and *Allobaculum* (Table 1). From our findings, it is evident that dosage size and administration days have a considerable impact on the diversity and relative abundance of gut microbiota. The gut microbiome acts as a modulator of host immune function, whose diversity and richness are affected by changeable factors, such as aging and sex, and constant factors, such as diet, medicinal therapies, and lifestyle [69,70].

As the composition of gut microbiota was changed by SiNPs, it was extremely correlated with a reduction in toxic effects, and SiNPs were safe to Balb/mice when administered intragastrically, especially the serum lipid levels, metabolic inflammation, and acid synthesis mice. It was speculated that liver function and renal function have no effect and individual groups have some effect on mice immunoglobulin and inflammatory cytokines, but most groups do not have significant differences; that is, SiNPs have a slight effect on the immune function of immunodeficient mice which were good at modulating gut microbiota, promoting the growth of some probiotics, and releasing some in addition. Fecal microbiota are still needed to provide direct evidence for this speculation. The comparative analysis, relationship, and difference between bacterial community composition, diversity, and structure were identified in past studies [56,61]. Factually, SiNPs caused innate immune responses at high doses in non-allergic mice. Individuals exposed to SiNPs might be more susceptible to manifesting allergic airway disease, due to the adjuvant-like properties of SiNPs [71]. Silica coating offered a potential solution to improve the biocompatibility of ZnO-NPs for applications such as antimicrobial agents in foods or food-associated products such as food packaging. However, high concentrations of silica-coated ZnO-NPs induced undesirable cytotoxicity to mammalian gut cells, and it was indicated that upstream is safer for nanotechnology and proved helpful in product development [72]. The levels of SCFAs and the composition of gut microbiota have indicated the relative abundance of *Lactobacillus* and *Bifidobacterium*, which might contribute to anti-obesity activity gut microbiota composition analysis at the phylum level, which showed that gut microbiota in mice mainly consist of *Firmicutes*, *Proteobacteria*, *Bacteroidetes*, *Verrucomicrobia*, and *Actinobacteria* [73]. Thus, diet has an impact on gut microbiota configuration, and nutritional disorders are related to a modification of the gut microbiota. Healthy and strong gut macrobiotics are critical for growth and weight gain, which shows that multifaceted macronutrient digestion by metabolizing SCFA influences the composition of the gut microbiota with specific changes to the major macronutrients contained in the diet. Age-related changes had an impact; the relative abundance of *Firmicutes* increased with age, and the relative abundance of *Bacteroides* decreased with age [74,75,76,77].

## 5. Conclusions

In summary, SiNPs showed no significant toxic effects on the cellular and hematological activities of immunodeficient mice. Furthermore, no immune dysfunction was found in the immunosuppressed mice groups after the administration of different levels of SiNPs. However, SiNPs significantly affect the abundance of different bacterial communities. LEfSe analysis revealed that SiNPs significantly increased the abundance of *Lactobacillus*, *Sphingomonas*, *Sutterella*, *Akkermansia*, and *Prevotella* and potentially reduced *Ruminococcus* and *Allobaculum*, which provides new insight into the regulation and administration of silica-based NPs. This would be useful for demonstrating the mechanism of action and predicting prospective SiNP effects. The effects of microbiome changes will be further investigated in some functional model of gut function, a function which might be anticipated to change with cyclophosphamide treatment. We are planning to do more research on the functional consequences of microbiome changes. In next investigations we will addressed this point that why we use an immunocompromised model and what effect of immunocompromised state is impinged on by the microbiome and NP mediated alterations.

## Figures and Tables

**Table 1:** The comparison of abundant intestinal bacteria and taxonomic relationships from immunodeficient mice treated with different levels of SiNPs.

**SiNP Treatment**	LDA Analysis of Mice Treated with Cy	LDA Analysis of Immunodeficient Mice Treated with Different Levels of SiNPs								
Comparison of gut microbiota after 6 days of SiNP treatment	CK6 vs. Cy6		Cy6 vs. Si-SL6		Cy6 vs. Si-SH6		Cy6 vs. Si-BL6		Cy6 vs. Si-BH6	
	Abundant genera		Abundant genera		Abundant genera		Abundant genera		Abundant genera	
	CK6 control group	Cy6-immunodeficient group	Cy6-immunodeficient group	Si-SL6-treated group	Cy6-immunodeficient group	Si-SH6-treated group	Cy6-immunodeficient group	Si-BL6-treated group	Cy6-immunodeficient group	Si-BH6-treated group
	*Prevotella,* *Anaerofustis*	*Oscillospira, Anaeroplasma, Allobaculum, Ruminococcus, Odoribacter, Sutterella, Coprococcus*	*Clostridium, Anaerofustis*	*Lactobacillus, Sphingomonas,* *Butyricimonas, Streptococcus*	*Ochrobactrum, Turicibacter, Dorea, Allobaculum*	*Coprobacillus, Sphingomonas*	*Clostridium, Staphylococcus, Desulfovibrio, Anaerovorax, Ochrobactrum, Allobaculum, Ruminococcus*	*Lactobacillus,* *Acinetobacter, Sphingomonas*	*Bacillus, Dehalobacterium, Anaeroplasma, Bilophila, Bifidobacterium, Anaerovorax, Serratia, Allobaculum, Ruminococcus, Oscillospira*	*Prevotella, Mycoplasma, Parabacteroides, Lactobacillus, Proteus, Mycobacterium, Sutterella, Paraprevotella, Coprobacillus, Turicibacter*
Comparison of gut microbiota after 12 days of SiNP treatment	CK12 vs. Cy12		Cy12 vs. Si-SL12		Cy12 vs. Si-SH12		Cy12 vs. Si-BL12		Cy12 vs. Si-BH12	
	Abundant genera		Abundant genera		Abundant genera		Abundant genera		Abundant genera	
	CK12 control group	Cy12-immunodeficient group	Cy12-immunodeficient group	Si-SL12-treated group	Cy12-immunodeficient group	Si-SH12-treated group	Cy12-immunodeficient group	Si-BL12-treated group	Cy12-immunodeficient group	Si-BH12-treated group
	*Streptococcus,* *Anaerostipes,* *Sutterella,* *Dorea,* *Bacteroides*	*Methylibium,* *Anaeromyxobacter,* *Desulfococcus,* *Sphingomonas,* *Thiobacillus,* *Aquicella*	*Dehalobacterium,* *Anaerofustis,* *Geothrix,* *Methylibium*	*Sutterella*	*Rhodoplanes,* *Lactobacillus,* *Geobacter,* *Kaistobacter,* *Syntrophobacter,* *Desulfococcus,* *Mycobacterium,* *Nitrospira,* *Candidatus Solibacter,* *Geothrix,* *Phenylobacterium,* *Thiobacillus,* *Sphingomonas,* *Anaeromyxobacter,* *Anaerolinea,* *Candidatus, Koribacter,* *Desulfobacca*	*Akkermansia*	*Streptococcus,* *Desulfovibrio,* *Anaeromyxobacter,* *Dehalobacterium,* *Nitrospira,* *Ruminococcus*	*Prevotella,* *Akkermansia,* *Sutterella*	*Anaerostipes,* *Dehalobacterium,* *Anaeromyxobacter,* *Geothrix,* *Methylibium,* *Geobacter,* *Ruminococcus,* *Mucispirillum*	*Prevotella,* *Sutterella,* *Akkermansia*

## Data Availability

Not applicable.

## Supplementary Materials

The following supporting information can be downloaded at: https://www.mdpi.com/article/10.3390/microorganisms11051183/s1, Figure S1. An effect of different levels of doses of SiNPs on the coefficient of immune and other major body organs: (a) weight of thymus; (b) weight of spleen; (c) thymus weight/body weight; (d) spleen weight/body weight; (e) kidney weight/body weight; (f) testis weight/body weight. Figure S2. Effects of SiNPs on blood indices, such as white blood cells (WBCs), lymphocytes, monocytes, neutrophils, platelet, and corpuscular hemoglobin: (a) number of WBCs; (b) number of lymphocytes; (c) number of monocytes; (d) number of neutrophils; (e) number of platelets; (f) mean corpuscular hemoglobin. Figure S3. The serum biochemical analysis showing the effects of SiNPs on serum profile in mice such as (a) alanine aminotransferase (ALT), (b) aspartate aminotransferase (AST), (c) alkaline phosphatase (ALP), (d) albumin (ALB), (e) total protein (TP), (f) creatinine (CREA), (g) urea/ blood urea nitrogen (BUN), and (h) uric acid (UA). Figure S4. Effects of silica nanoparticles on inflammatory cytokines; (a) TNF-α, (b) IL-1β, and (c) IL-2 in the ileum. Figure S5. LEfSe analysis showing intestinal bacteria that were significantly different in abundance between the CK6 control group and the Cy6-immunodeficient group (a and b) before SiNP treatment and (c and d) between the Cy6-immunodeficient group and the Si-BH6-treated group after 6 days of treatment with SiNPs. Figure S6. LEfSe analysis demonstrating the comparison of intestinal bacterial genera that were significantly different in abundance between the control group (CK12) and the immunodeficient group (Cy12) (a and b) before SiNP treatment and the Cy12-immunodeficient group and the Si-BL12-treated group (c and d) and Si-BH12-treated group (e and f) after 12 days of treatment with SiNPs.
