# Supplementary material for: Toxicity and Impact of Silica Nanoparticles on the Configuration of Gut Microbiota in Immunodeficient Mice
Source: Microorganisms. 2023 Apr 30;11(5):1183. doi: 10.3390/microorganisms11051183 (PMC10222337; doi:10.3390/microorganisms11051183)
Supplement: Supplementary file 1 [file microorganisms-11-01183-s001.zip › microorganisms-2294153-Supplementary.pdf]

Supplementary Materials

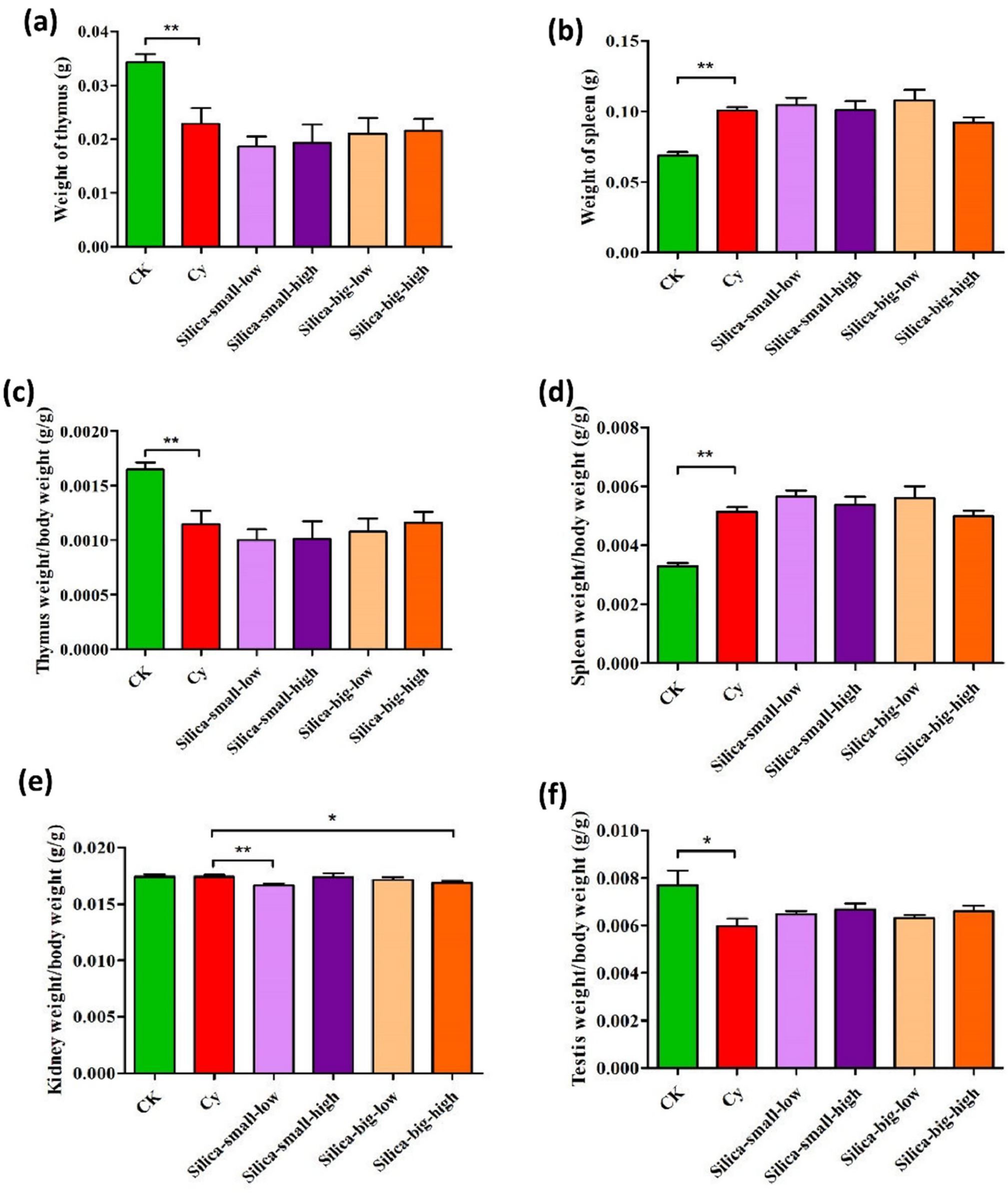

Figure S1. An effect of different level of doses of SiNPs on immune and other major body organs coefficient: **(a)** weight of thymus; **(b)** weight of spleen; **(c)** Thymus weight/body weight; **(d)** spleen weight/body weight; **(e)** kidney weight/body weight; **(f)** Testis weight/body weight.

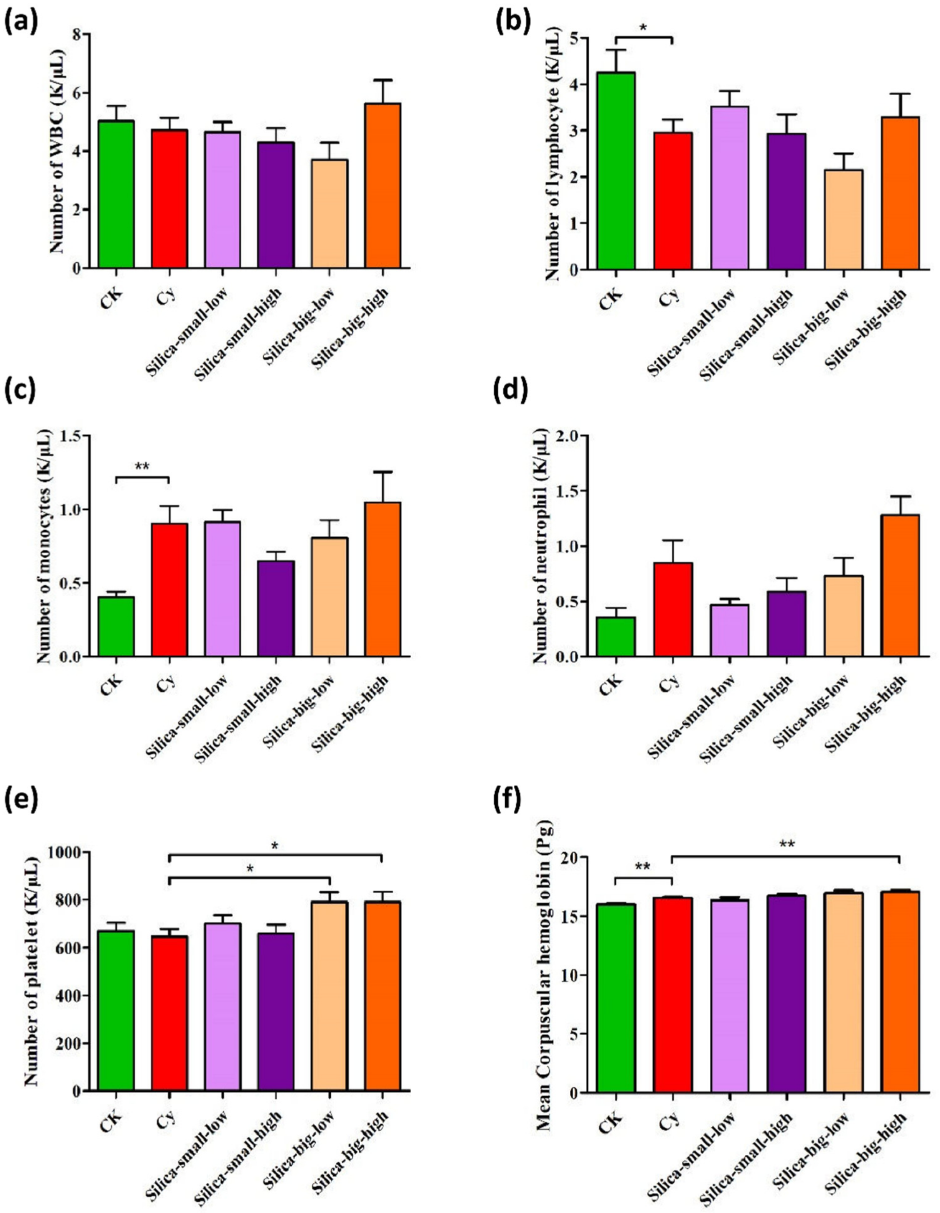

Figure S2. Effects of SiNPs on blood indices, such as white blood cells (WBCs), lymphocyte, monocytes, neutrophils, platelet, and corpuscular hemoglobin: **(a)** number of WBC; **(b)** number of lymphocytes; **(c)** number of monocytest; **(d)** number of neutrophil; **(e)** number of platelets; **(f)** mean corpuscular hemoglobin.

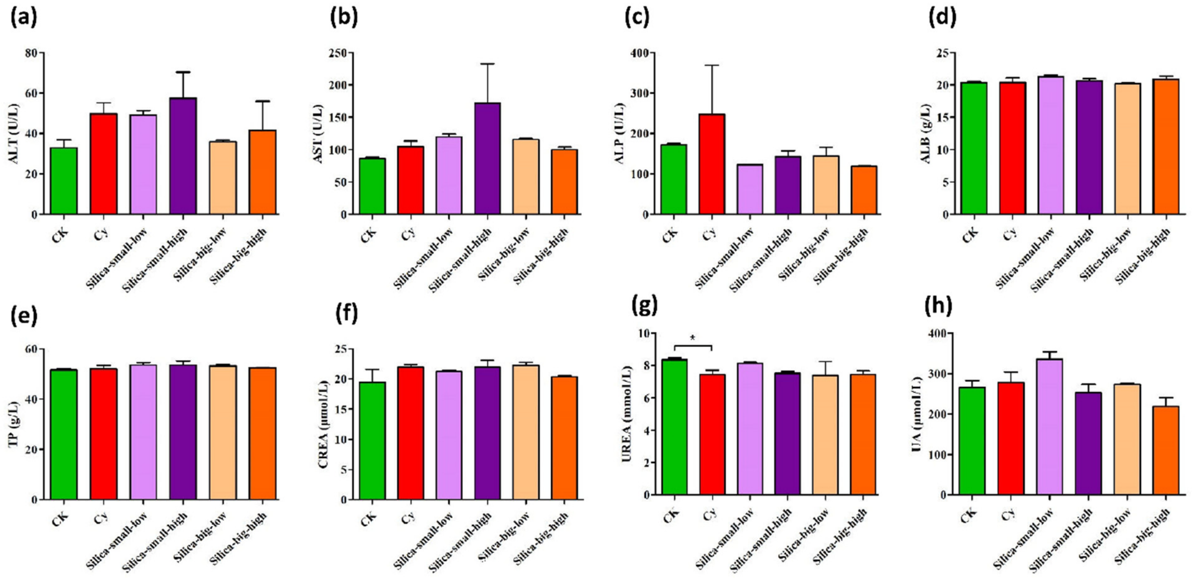

Figure S3. The serum biochemical analysis showing the effects of SiNPs on serum profile in mice such as **(a)** alanine aminotransferase (ALT), **(b)** aspartate aminotransferase (AST), **(c)** alkaline phosphatase (ALP), **(d)** albumin (ALB), **(e)** total protein (TP), **(f)** creatinine (CREA), **(g)** urea/ blood urea nitrogen (BUN), and **(h)** uric acid (UA).

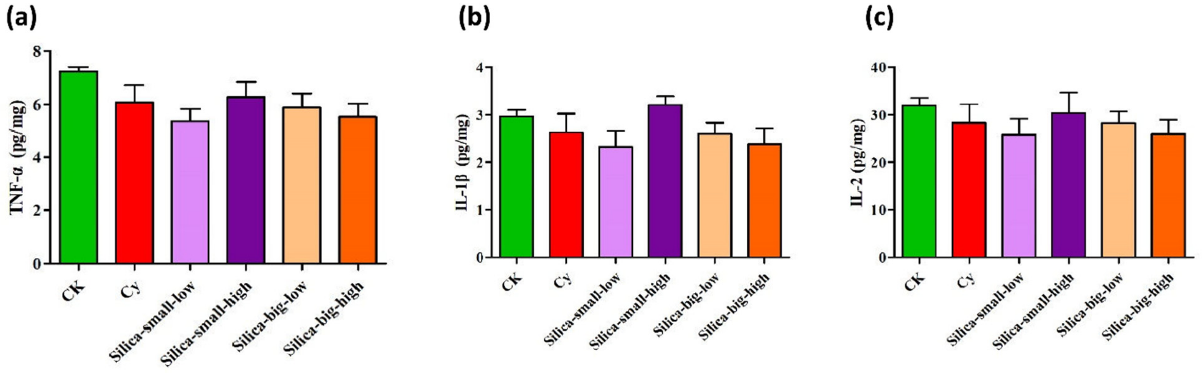

Figure S4. Effects of silica nanoparticles on inflammatory cytokines; **(a)** TNF-α**, (b)** IL-1β, and **(c)** IL-2 in ileum.

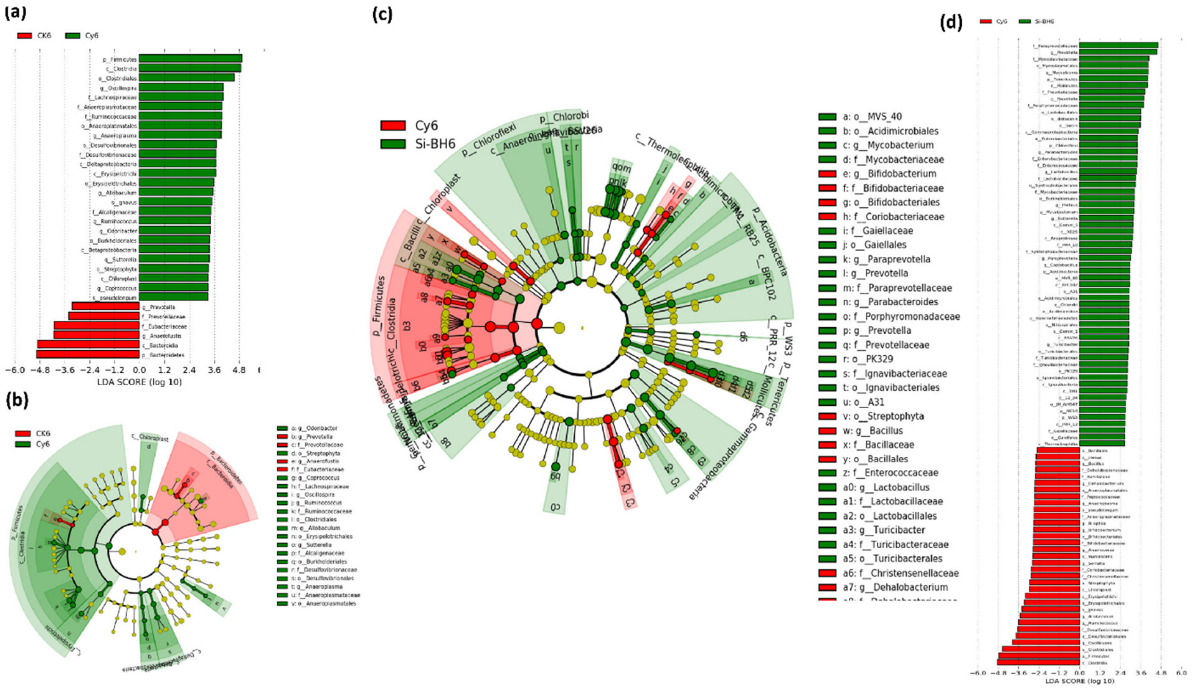

Figure S5. LEfSe analysis showing intestinal bacteria that were significantly different in abundance between the CK6 control group and the Cy6-immunodeficient group **(a** and **b**) before SiNP treatment and **(c** and **d)** between the Cy6-immunodeficient group and the Si-BH6-treated group after 6 days of treatment with SiNPs.

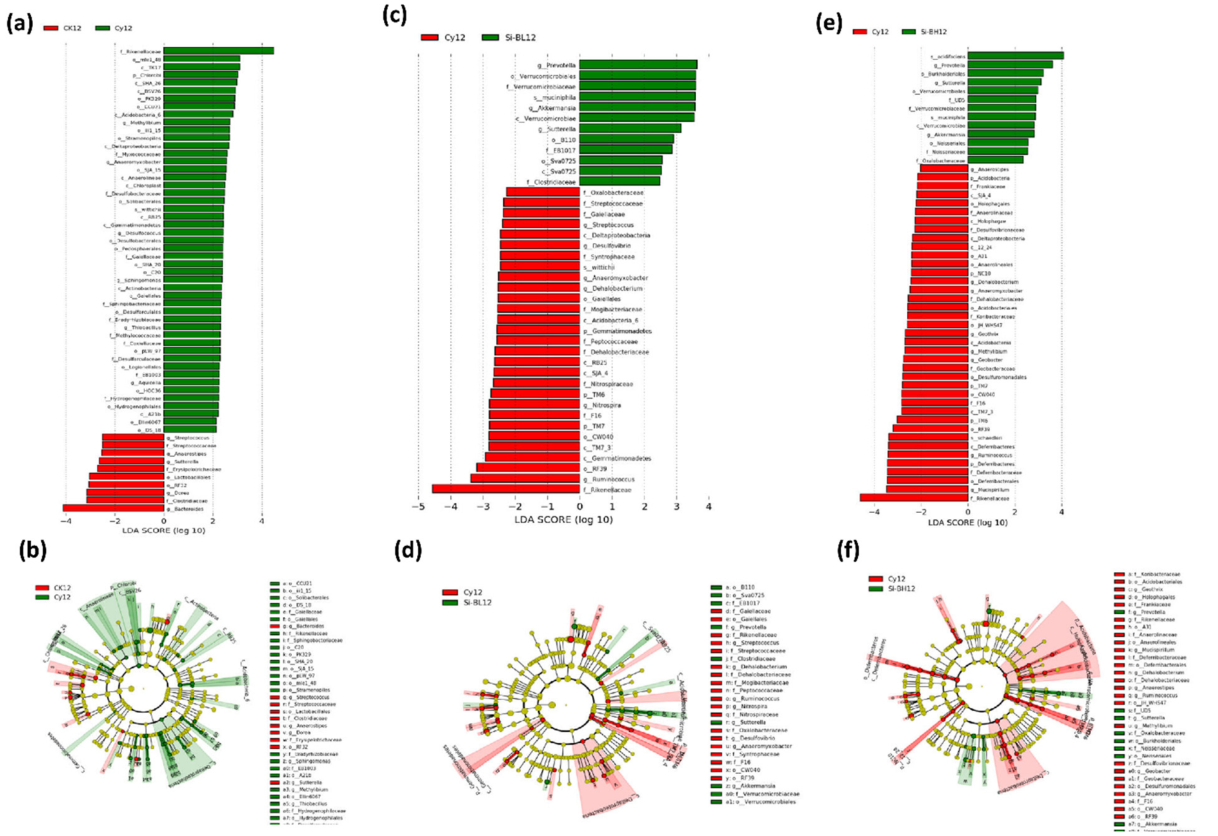

Figure S6. LEfSe analysis demonstrating the comparison of intestinal bacterial genera that were significantly different in abundance between the control group (CK12) and the immunodeficient group (Cy12) (**a** and **b**) before SiNP treatment and the Cy12-immunodeficient group and the Si-BL12-treated group (**c** and **d**) and Si-BH12- treated group (**e** and **f)** after 12 days of treatment with SiNPs.
